# RETRACTION: 1,25‐Dihydroxyvitamin D exerts an antiaging role by activation of Nrf2‐antioxidant signaling and inactivation of p16/p53‐senescence signaling

**DOI:** 10.1111/acel.14383

**Published:** 2024-10-18

## Abstract

**RETRACTION**: Chen, L., Yang, R., Qiao, W., Zhang, W., Chen, J., Mao, L., Goltzman, D., Miao, D. (2019). 1,25‐Dihydroxyvitamin D exerts an antiaging role by activation of Nrf2‐antioxidant signaling and inactivation of p16/p53‐senescence signaling. *Aging Cell*, 18(3), e12951. https://doi.org/10.1111/acel.12951

The above article, published online on 24 March 2019 in Wiley Online Library (wileyonlinelibrary.com), has been retracted by agreement between; the journal Editor‐in‐Chief, Monty Montano; The Anatomical Society; and John Wiley & Sons Ltd. The retraction has been agreed due to duplications observed between elements of Figures 1h and 1j; 5d and 5f; 3a and 6j; and Supplemental Figures 4c and 4e.

The authors acknowledged their errors in figure management, which led to the duplications observed between Figures 3a and 6j, as well as between S4c and S4e. They also admitted to copying and pasting small areas from figure S4e into the same image for aesthetic purposes. Additionally, they admitted to editing Figure 4d (ChIP gel image) to reduce noise and enhance the clarity of the bands shown. The authors provided some data and an explanation for the similarities observed between Figures 1h and 1j; and Figures 5d and 5f. However, their explanation was not sufficient. Due to the extent of the identified issues, the editors have lost confidence in the data presented. The authors disagree with the retraction.

**RETRACTION**: Chen, L., Yang, R., Qiao, W., Zhang, W., Chen, J., Mao, L., Goltzman, D., Miao, D. (2019). 1,25‐Dihydroxyvitamin D exerts an antiaging role by activation of Nrf2‐antioxidant signaling and inactivation of p16/p53‐senescence signaling. *Aging Cell*, 18(3), e12951. https://doi.org/10.1111/acel.12951

The above article, published online on 24 March 2019 in Wiley Online Library (wileyonlinelibrary.com), has been retracted by agreement between; the journal Editor‐in‐Chief, Monty Montano; The Anatomical Society; and John Wiley & Sons Ltd. The retraction has been agreed due to duplications observed between elements of Figures 1h and 1j; 5d and 5f; 3a and 6j; and Supplemental Figures 4c and 4e.

The authors acknowledged their errors in figure management, which led to the duplications observed between Figures 3a and 6j, as well as between S4c and S4e. They also admitted to copying and pasting small areas from figure S4e into the same image for aesthetic purposes. Additionally, they admitted to editing Figure 4d (ChIP gel image) to reduce noise and enhance the clarity of the bands shown. The authors provided some data and an explanation for the similarities observed between Figures 1h and 1j; and Figures 5d and 5f. However, their explanation was not sufficient. Due to the extent of the identified issues, the editors have lost confidence in the data presented. The authors disagree with the retraction.

